# Retinal Organoids: Cultivation, Differentiation, and Transplantation

**DOI:** 10.3389/fncel.2021.638439

**Published:** 2021-06-28

**Authors:** Xuying Li, Li Zhang, Fei Tang, Xin Wei

**Affiliations:** ^1^Department of Ophthalmology, West China Hospital, Sichuan University, Chengdu, China; ^2^Department of Ophthalmology, Shangjin Nanfu Hospital, Chengdu, China

**Keywords:** retinal organoid, stem cell, retinal ganglion cell, photoreceptor cell, replacement therapy

## Abstract

Retinal organoids (ROs), which are derived from stem cells, can automatically form three-dimensional laminar structures that include all cell types and the ultrastructure of the retina. Therefore, they are highly similar to the retinal structure in the human body. The development of organoids has been a great technological breakthrough in the fields of transplantation therapy and disease modeling. However, the translation of RO applications into medical practice still has various deficiencies at the current stage, including the long culture process, insufficient yield, and great heterogeneity among ROs produced under different conditions. Nevertheless, many technological breakthroughs have been made in transplanting ROs for treatment of diseases such as retinal degeneration. This review discusses recent advances in the development of ROs, improvements of the culture protocol, and the latest developments in RO replacement therapy techniques.

## Introduction

An organoid is a type of tissue induced from stem cells that is able to automatically form three-dimensional structures with a variety of cell types. Organoid structures are highly similar to the organ structures in the human body (Khan et al., 2016). At present, the types of brain organoids that can be induced include the hippocampus, pituitary gland, forebrain, and retina (Brawner et al., 2017; Eastlake et al., 2019; Hua et al., 2020). Retinal organoids (ROs) are induced from stem cells and develop into the optic vesicle and optic cups, which are finally compressed, thinned, and matured. Mature ROs have all of the cell types and structures included in the retina (Miltner and La Torre, 2019). Currently, human embryonic stem cells, induced pluripotent stem cells (iPSCs), retinal progenitor cells (RPCs), mesenchymal stem cells, and other types of stem cells have been used to cultivate ROs with multi-cell retinal laminar structure and ultrastructure (Capowski et al., 2019).

The development of ROs is a major technological breakthrough in the field of transplantation therapy. Because ROs are highly similar to the retinal structure and include all of the cell types of the human retina, the corresponding tissue slices can be separated from the cultured organoids and transplanted into the diseased layers of the retina. This functional replacement also preserves the original structure of the retina, overcoming the disadvantage of previous single cell type transplantation techniques (Singh et al., 2018; Ahmad et al., 2020). Some studies have shown that ROs provide an opportunity to restore vision in patients with advanced retinal degeneration (McLelland et al., 2018; Ahmad et al., 2020).

Compared with two-dimensional stem cell techniques, three-dimensional ROs can more realistically simulate the interaction between the micro-environment in each layer *in vivo*, which allows investigation of the poor efficacy of some drugs in practical applications and may therefore result in the development of more efficient drugs. Additionally, the three-dimensional suspension medium is beneficial for generation of certain cell types, such as neurons, thus providing the possibility to improve the production efficiency of retinal ganglion cells (RGCs) and obtain longer axons. This is particularly beneficial for the study of the pathogenesis and repair of neuro-degenerative diseases such as glaucoma (Wright et al., 2015). Goureau et al. (2020) induced and cultured iPSCs from blind patients and obtained ROs with pathological characteristics, in which early loss of photoreceptor cells (PRCs) could be observed.

However, the use of ROs for disease modeling and even clinical applications still has various deficiencies at the current stage. These limitations mainly include the long culture process, insufficient yield, and great heterogeneity among ROs cultivated by different protocols. Meanwhile, research on various diseases requires differentiation of ROs to produce sufficient numbers of specific cells.

This review focuses on the development process of ROs, improvements in the culture protocol of ROs, and the induction of differentiation of PRCs and RGCs in ROs to provide useful information about disease modeling and clinical applications of ROs.

## Development of Retinal Organoids

The development of ROs occurs as follows. First, stem cells proliferate and aggregate and are then induced to form neuroepithelial cells. Then, the cells develop into neuro–spheres, including forebrain progenitor cells and optic vesicles. The latter will develop into optic cups and the neural retina, and the cells will differentiate into RGCs, amacrine cells and horizontal cells, followed by PRCs (including rods and cones), as well as bipolar cells and Muller glial cells (Reichman et al., 2014).

The differentiated cells spontaneously undergo nuclear migration, form a spire-like shape, and finally arrange into a laminated structure, where the RGCs are located in the inner layer and the PRCs are located in the outer layer of ROs (Nakano et al., 2012; Ohlemacher et al., 2015, 2019; Cui et al., 2020; Fligor et al., 2020). At the same time, the mature PRCs in the organoids have an outer segment and photosensitivity, which can be used in the study of retinal degeneration and other related diseases (Zhong et al., 2014).

Eiraku et al. (2011) showed that this migration is determined by intracellular processes, such as expression of high levels of myosin at the initial stage of organoid invagination, resulting in a certain degree of rigidity, and this process is related to the specific local regulation of the epithelium. Phillips et al. also found that the human–derived retina expresses intercellular communication genes such as *CX36* at this stage. Meanwhile, the genes expressed, such as *VGLUT1*, *SNAP-25*, *AMPA1*, *NMDA2A*, and *MGLUR6*, are also closely related to the formation of synaptic connections, and the platelet reactive protein gene *THBS2* is up-regulated (Phillips et al., 2012; Palomo et al., 2014).

**FIGURE 1:**
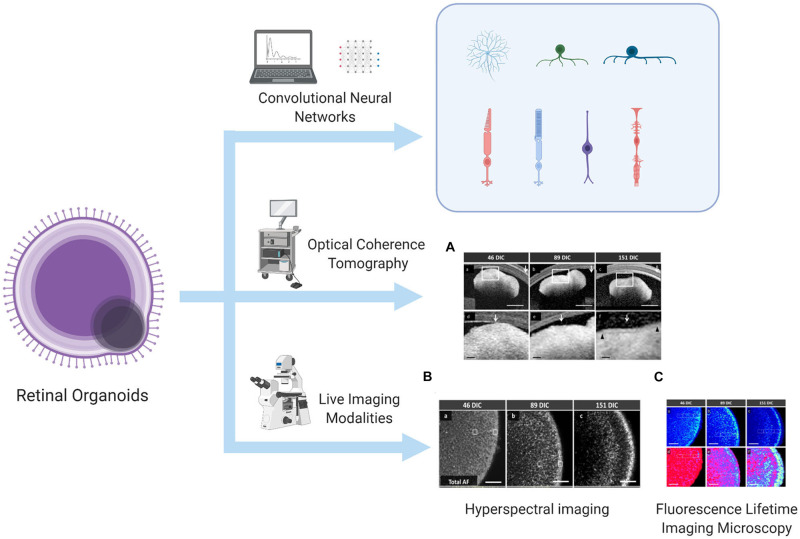
Algorithm to predict and identify organoid differentiation, and real-time imaging modalities to monitor the metabolic status of the lamellar structure of ROs. This illustration is created by Biorender.com **(A)** OCT image, **(B)** HSpec image, and **(C)** FLIM image are reprinted with permission from ref (Browne et al., 2017). Copyright © 2017 Browne AW. et al.

Currently, the development and differentiation process of ROs can be continuously monitored and quantitatively evaluated. Some studies have developed an algorithm based on deep learning using bright-field images, allowing researchers to predict the direction of differentiation and identify differentiation even before gene expression in the mouse–derived organoid culture (Kegeles et al., 2020a). When human–derived organoids develop into a lamellar structure, real-time imaging modalities such as fluorescence lifetime imaging microscopy, hyperspectral imaging, and optical coherence tomography can be used to monitor the metabolic status of ROs and even PRCs, and these methods will not damage the culture structure (Browne et al., 2017). The above-mentioned technologies that can be used to evaluate and monitor the development and differentiation of ROs have been summarized in Figure 1. Immunocytochemistry methods have also been used to monitor typical genes such as *VSX2* (corresponding to RPCs), *HuC/D* (corresponding to RGCs), *CHAT* (corresponding to starburst amacrine cells), *Crx* (corresponding to PRCs), and *RXR* (corresponding to cone precursors), aiming to observe and map the progression of differentiation of each human–derived cell type (Chichagova et al., 2019). The improved protocols mentioned in this chapter are summarized in Table 1.

**TABLE 1:** Summary of improved protocols of retinal organoids.

Study	Improved protocols	Effects
**Development of retinal organoids**		
Kegeles et al., 2020a	An algorithm based on deep learning using bright-field images	Predicting the direction of differentiation and identify differentiation
Browne et al., 2017	Real-time imaging modalities including fluorescence lifetime imaging microscopy, hyperspectral imaging, and optical coherence tomography	Non-invasive real-time monitoring of metabolic status of ROs and even PRCs
Chichagova et al., 2019	Immunocytochemistry methods	Observing and mapping the progression of differentiation
**Promotion of retinal organoid differentiation**		
Llonch et al., 2018	Adding mESC to a serum-free 96-well plate to keep the cell density at 3000/well, then adding extracellular matrix after induction of embryoid bodies	Differentiating the culture into rigid neuroepithelium, express PAX6 after 1 week, and differentiate into optic vesicles
Zhu et al., 2018	Stem cells were manually separated after 2–4 weeks to form rosette-like aggregates and were cultured in a low-adhesion 6-well culture plate and NSC medium which was changed every 2–3 times a week.	Promoting the differentiation of stem cells into organoids with an optic cup shape and multi-layered cell structure.
Ohlemacher et al., 2015	Human iPSCs are induced into RPCs by neural induction medium and retinal differentiation medium, and then remain suspended in the neural induction medium to form aggregates.	Inducing hPSC into RPCs and visual vesicles
Reichman et al., 2017	Passing human iPSC five times in Essential 8	Skipping the embryoid body stage and directly differentiating into RPCs and neuroretinal structures, shortening the time from 6 months to 1 month.
Perepelkina et al., 2019	Using Synthemax SC II (vitronectin mimic peptide) at a concentration of 0.005–0.02 mg/ml to induce early differentiation of the retina	Achieving xenogeneic-free cell culture without the dose-dependent effects
Chen et al., 2019	Polybenzyl glutamate scaffold	Promoting the increase in RGCs and axon growth in ROs.
Kador et al., 2013	Electrospun scaffold	Promoting the survival of RGC and guide axons to project radially along the scaffold
Kador et al., 2014	Creating the concentration gradient of Netrin-1 on the electrospun scaffold	Promoting the polarization of RGC cells
Yao et al., 2015	Subretinal space transplantation of mRPC with PCL stent	Guiding stem cells to differentiate, help cells move and express corresponding markers
**Augmentation of retinal organoid production**		
Zhu et al., 2018	Passing the aggregates in the ratio of 1:3, and use low-adhesion culture plates and NSC medium for the neuroepithelium	Increasing the yield of ROs
Völkner et al., 2016, 2019	Induceing mESC differentiation with Matrigel. The neuroepithelium is separated at the ratio of 1:3, and 40% oxygen is added before maturity.	Increasing the yield of ROs
Regent et al., 2020	Dispersing the adherent cell clusters and keep them in suspension	Increasing the yield of ROs by 5 times
Perepelkina et al., 2019	Adding 2% lipid concentrate or 0.06% methyl cellulose to the medium	Reducing the aggregation of culture.
**Reduction of retinal organoid heterogeneity**		
Llonch et al., 2018	Adding mESC to a serum-free 96-well plate to keep the cell density per well of 96-well plate at 10000/well	Reducing heterogeneity and increase production
Zhu et al., 2018	RO culture staging system, including light microscopy, electron microscopy, optical coherence tomography, metabolic imaging, immunohistochemistry, and other technologies.	Synchronizing the cultivation phase and reduce heterogeneity
**Extraction and transplantation of ganglion cells**		
Wang et al., 2019	Transplanting RPC in RO to the ganglion cell layer	Making up for the lack of RGC in the ganglion cell layer
Rabesandratana et al., 2020	Intravitreal injection of RGC screened by cell marker THY1	Contacting the remaining RGCs
Tanaka et al., 2015	BDNF transforms organoids into the two-dimensional development model after developing into the three-dimensional vesicle structure	Increasing the differentiation efficiency of ganglion cells and making them have higher-level functions
**Extraction and transplantation of photoreceptor cells**		
Chen et al., 2016	The High Efficiency Hypoxia Induced Generation of Photoreceptors in Retinal Organoids protocol	Restoring the development of photoreceptor cells *in vivo*
Pan et al., 2020	The reporter gene tdTomato	Non-invasive monitoring of photoreceptor cell differentiation

## Improvement of Retinal Organoid Culture

### Promotion of Retinal Organoid Differentiation

The culture process of ROs is very time-consuming. According to reports, at least 100 days are required for human pluripotent stem cells to form ROs and differentiate into mature and functional PRCs (Gasparini et al., 2019). Therefore, promotion of the differentiation of organoids to shorten the culture time is an important issue. Cells expressing *PAX6* have been shown to be the major cell type comprising ROs. In one study, mouse embryonic stem cells were added to serum-free 96-well plates with approximately 3,000 floating cells in each well to induce clumped embryoids. Then, extracellular matrix was added to the culture medium to differentiate the culture into neuroepithelial cells with a degree of rigidity. After a week, transcription factors specific to the eye such as *PAX6* were expressed, and differentiation into optic vesicles began (Llonch et al., 2018). Zhu et al. attempted to induce iPSC NCL-1 produced by CD34(+) umbilical cord blood cells to produce rosette-like nerve aggregates by culturing cells for 2–4 weeks and then manually separating the cells and switching to ultra-low adhesion 6-well plates with neural stem cells medium. Changing the medium 2–3 times a week can induce the aggregates to self-organize and differentiate into organoids. The organoids also have a laminated cell structure and take the shape of the optic cup (Zhu et al., 2018). For the induction culture of human pluripotent stem cells, the stem cells need to be suspended in neural induction medium to form aggregates and differentiate into neuroepithelium. After 2 weeks, the aggregates are cultured in retinal differentiation medium to induce their differentiation into three-dimensional optical vesicles (Ohlemacher et al., 2015). However, the xenogeneic/feeder-free culture protocol can greatly shorten the culture time. Reichman et al. used Essential 8 medium, which is a pure chemical medium, to induce human iPSCs to differentiate into RPCs and neuroretinal structures after five passages, bypassing the embryoid body stage and thus shortening the culture time from 6 months to 1 month. Additionally, this process can extend the lifespan of PRCs that are functional in organoids for up to 10 months (Reichman et al., 2017). There is another protocol achieving a xenogeneic-free cell culture. Perepelkina et al. suggested that a low concentration of Synthemax SC II (vitronectin mimic peptide) can be used to replace the Matrigel medium to induce the early differentiation of the mouse ESC–derived retina. Furthermore, Synthemax SC II concentrations in the range of 0.005–0.02 mg/ml have no dose-dependent effects on the cells (Perepelkina et al., 2019).

It is currently thought that insulin-like growth factor 1, retinoic acid, and triiodo thyroxine not only play an important role in the development of actual retinas, but are also essential for cell differentiation in ROs. Studies have also found that adding serum and high glucose concentrations to the medium promotes the differentiation and development of human iPSC–derived ROs (Chichagova et al., 2019; Slembrouck-Brec et al., 2019).

In addition, an electrospinning polybenzyl glutamate scaffold is also used. This material is composed of polypeptides containing glutamate. Because the glutamic acid pathway is related to the development of neurons, it promotes an increase in RGCs and axon growth in human iPSC–derived Ros. Moreover, compared with ordinary culture medium, hiPSC on PBG takes a shorter time to form organoids and differentiate into RGC (Chen et al., 2019). Previous studies have designed the electrospun scaffold, which can simulate the growth process of RGC axons *in vivo*, thereby promoting the survival of RGCs and guiding the axons to project along the radial direction of the scaffold (Kador et al., 2013). Kador et al. (2014) designed the concentration gradient of Netrin-1, a guidance factor, on the electrospun scaffold to promote the polarization of RGCs, and further promote the connection of RGC and visual pathway during stem cell transplantation. Studies have also reported that the use of polycaprolactone scaffold for mouse RPC subretinal space transplantation is able to guide stem cell differentiation and help cells move to the outer nuclear layer of the retina, and express photoreceptor-related markers. These strategies may provide references for promoting the effective transplantation of ROs (Yao et al., 2015).

In summary, maintaining a certain cell density and keeping the cells in suspension can induce the formation of embryoid body, and the addition of extracellular matrix promotes the rigidity of the culture. When high-quality iPSCs are suspended, rosette-like nerve aggregates will appear first, and they can differentiate into ROs after being induced by neural stem cells medium. And hPSC can be cultured by neural induction medium and retinal differentiation medium to produce optical vesicles. The xenogeneic-free cell culture protocols such as Essential 8 medium and low-concentration Synthemax SC II medium can bypass the embryoid body stage and greatly shorten the time required for differentiation. In addition, electrospun scaffolds can promote RGC axon projection, and glutamate-containing scaffold can shorten the differentiation time of RGCs, and the Netrin-1 protein gradient can increase the polarization ratio of RGCs. There are also scaffolds such as polycaprolactone scaffold, which can promote the movement of the culture to the corresponding level of the retina, thereby providing the possibility for precise replacement therapy. The improved protocols mentioned in this section are summarized in Table 1.

### Augmentation of Retinal Organoid Production

One of the major problems within RO research is the insufficient yield of organoid culture. In suspension, stem cells tend to aggregate, resulting in low organoid yields. At present, most studies suggest separating organoids manually to improve production efficiency (Völkner et al., 2016). Zhu et al. (2018) attempted to improve the production efficiency of human–derived ROs by screening high-quality iPSC NCL-1, subculturing the cells at 1:3 when aggregation is observed, and isolating and cultivating the neuroretina part in neural stem cell medium in a low-adhesion culture plate. Some studies have explored protocols to produce large-scale laminated organoids. In contrast to the mainstream protocol, this method does not require separation of aggregates, but instead induces the aggregated mouse embryonic stem cells to form neurons using Matrigel. The resulting neuroepithelium is then separated into three equal-sized parts, and 40% oxygen is added when the neuroepithelium nears maturity. This solution can double the production of organoids and improve their quality (Völkner et al., 2016, 2019). In addition to separating aggregates, some studies have explored a different method to increase yield, that is, promoting the development and differentiation of cultures that have not yet formed the three-dimensional structure of organoids. Regent et al. extracted and dispersed human–derived cell clusters that were not suspended in medium with a cell scraper, and the small clumps were kept floating. 24 h later, a large number of ROs shaped like neuroepithelium could be observed. This method increased the yield by five times, and no significant difference was observed compared with ROs cultured by dissection and separation (Regent et al., 2020). This finding indicates that the yield of the cultures is related to the degree of culture suspension. Perepelkina et al. (2019) suggested that adding 2% lipid concentrate or 0.06% methyl cellulose to the medium can reduce aggregation of the culture, thus improving the mouse–derived culture efficiency.

In short, the organoid production is closely related to the degree of suspension of the culture. Manual separation of aggregates or neuroepithelium is the most direct solution to increase organoid production. Reducing the degree of cell aggregation or suspending adherent cells is another strategy to increase yield. The improved protocols mentioned in this section are summarized in Table 1.

### Reduction of Retinal Organoid Heterogeneity

In addition to the low yield of organoid cultures, another obstacle to organoid research lies in the great heterogeneity among various organoid cultures. Therefore, it is urgent to explore a reproducible culture protocol to reduce heterogeneity. This is also a major problem that hinders the translation of RO research into clinical practice and leads to the risk of damaging the normal retinal structure of the recipient and causing teratoma when retinal organ transplantation is performed clinically (Ahmad et al., 2020). Although studies have shown that, for ROs derived from reprogrammed human iPSCs, the culture process itself may enable the ROs to acquire immunogenicity and cause the recipient to produce a T-cell immune response in the transplantation stage, thus avoiding the risk of teratomas caused by organoid heterogeneity (Phillips et al., 2012). Studies that have summarized the culture schemes that promote RO differentiation have shown that the shape of the well, cell number in each well, composition of each medium, and method of culturing aggregates are factors that lead to the great heterogeneity among ROs in different culture conditions. When the mouse ESC–derived cell number is 10,000/well in a 96-well plate, the size of the aggregates generated will be 380–550 μm, which will significantly reduce the degree of heterogeneity of organoids among different batches and greatly increase the number of organoids at the same time (Perepelkina et al., 2019).

Capowski et al. developed a human–derived RO culture staging system using light microscopy, optical coherence tomography, metabolic imaging, electron microscopy, immunohistochemistry, and other technologies to synchronize various culture stages and reduce organoid heterogeneity. First, the organoids were divided into the neuroepithelial stage, dark nucleus stage, and hairlike surface appendage stage using light microscopy. Optical coherence tomography scanning of these three culture stages revealed that no layered structures were present in stage 1 and 2, and alternating high and low reflectivity layers appeared in stage 3. Immunohistochemistry results showed that the main cells in stage 1 were neural RPCs, RGCs, and starburst amacrine cells. A large number of PRC precursors and gradually maturing cone and rod cells appeared in stage 2. Stage 3 included more mature outer nuclear layer and plexiform layer cells, as well as mature upper and inner core layer cells. At this time, PRCs already have light-sensing function, but the cells formed in stage 1 exhibit structural disorder and degeneration (Capowski et al., 2019).

In a nutshell, maintaining the consistency of cell density in each culture dish can reduce the heterogeneity of organoids. The use of light microscopy, electron microscopy, optical coherence tomography and other methods can synchronize the differentiation process of cultures, thereby reducing the heterogeneity between products. The improved protocols mentioned in this section are summarized in Table 1.

## Extraction and Transplantation of Various Cell Types in the Retina

### Extraction and Transplantation of Ganglion Cells

Ganglion cells are closely related to glaucoma. Diseases that can cause glaucoma may lead to the loss of ganglion cells through mechanical compression, abnormal blood supply, and immune mechanisms (Cohen and Pasquale, 2014). Wang et al. attempted to transplant RPCs in human–derived ROs into the ganglion cell layer of mice, but only a portion of RGCs remained in this layer. The implanted RPCs filled the original positions of RGCs in the ganglion cell layer and even expressed the RGC-specific marker BRN3A (Wang et al., 2019). Rabesandratana et al. also reported that the cell marker THY1 can be used to screen out effective human-derived RGCs. When these cells are injected into recipient mice vitreous, they will associate with the remaining RGCs and survive for 1 month (Rabesandratana et al., 2020).

Unfortunately, the number of ganglion cells in organoids is not substantial, and they will gradually degenerate over time (Pereiro et al., 2020). In ROs derived from PSCs of Thy1-EGFP transgenic mice, the ganglion cell markers BRN3B and SMI-312 can be maintained for 2–3 weeks, and the RO ganglion cells obtained from human iPSCs can be identified for 4 weeks (Kobayashi et al., 2018), but the expression time is still much shorter than that of PRCs. Miltner and La Torre (2019) suggested that ganglion cell apoptosis may be mediated by the Bax signaling pathway. Therefore, to study RGCs in ROs, it is necessary to explore a protocol that can efficiently produce RGCs in large numbers. Converting three-dimensional organoid cultures into two-dimensional cultures is an effective method to amplify ganglion cells. Tanaka et al. found that brain-derived neurotrophic factor can transform human–derived organoids into a two-dimensional development model after the formation of three-dimensional optic vesicle structures, thereby increasing the differentiation efficiency of ganglion cells. The differentiation rate was as high as 90%, and the cells expressed BRN3B, MATH5, and other specific markers. The cells also had characteristics of ganglion and nerve cells, such as axoplasmic transport and action potentials, suggesting that these cells have a higher level of function (Tanaka et al., 2015).

A study by Fligor et al. also suggested that another factor that influences the effect of RGC transplantation is the length of RGC axons. An insufficient RGC axon length will affect the synaptic connections between ganglion cells and the visual pathway, leading to failure of RGC transplantation. Thus, investigation of factors affecting the length of ganglion cell axons in organoids is conducive to the improvement of RGC replacement therapy (Fligor et al., 2018). Prolonging the survival time of ganglion cells is another strategy for RGC replacement. Kobayashi et al. (2018) found that ganglion cells extracted from more mature organoids have a greater ability to elongate axons, allowing easier establishment of connections with the brain. Aparicio et al. found that human–derived RPCs and ganglion cell precursors express high levels of CD184 markers, while mature ganglion cells mainly express CD171. Therefore, this differential expression of ganglion cell markers allows monitoring of ganglion cell development and collection of cells at specific times to obtain ganglion cells that can produce sufficiently long axons (Aparicio et al., 2017). Tanaka et al. (2015) found that addition of Muller cells to human–derived ganglion cell medium can significantly improve the survival rate of ganglion cells and significantly extend their functional axons.

Injection of Muller glial cells derived from ROs into rats with NMDA-depleted RGCs could partially restore vision, which suggested that transplantation of Muller cells might be helpful to repair retinal degeneration and vision loss caused by RGC degeneration (Eastlake et al., 2019). Pereiro et al. (2020) suggested that this process is related to up-regulation of the transcription factor *ATOH7*, which is required for mouse–derived ganglion cell development, in Muller cells. However, over-expression of *ATOH7* during mouse–derived RGC formation also correspondingly reduced the number of remaining progenitor cells, resulting in a decrease in the number of PRCs, which develop later than ganglion cells (Zhang et al., 2018).

In conclusion, the RGC of RO can be connected with the remaining ganglion cells in the diseased retina. However, the low yield of RGC and the inability to obtain axons of sufficient length are currently obstacles to clinical transformation. Two-dimensional expansion of RGC through brain-derived neurotrophic factor is a feasible strategy to solve the problem of low yield; screening and purifying mature RGC from RO for transplantation, adding Muller cells to the culture medium to promote the development of ganglion cells is a potential strategy to solve the problem of insufficient axon length. The improved protocols and research findings mentioned in this section are separately summarized in Tables 1, 2.

**TABLE 2:** Summary of findings related to retinal organoid culture process and transplantation.

**Study**	**Findings**
**Retinal ganglion cells**	
Kobayashi et al., 2018	Mature ganglion cells are more capable of extending axons.
Aparicio et al., 2017	Ganglion cell precursors express CD184. Mature ganglion cells express CD171.
Tanaka et al., 2015	Muller cells promote ganglion cells to survive and extend axons.
Eastlake et al., 2019	Transplanting Muller cells conduces to repair RGC degeneration-related diseases.
**Photoreceptor cells**	
Singh and Nasonkin, 2020	Organoid lamellar transplantation compensates for degraded photoreceptor cells. Transplanting PR and neurons together can increase the survival rate of neurons.
Pearson et al., 2012	Organoid grafts provides dark vision.
McLelland et al., 2018	The connection with the IPL of the receptor indicates the synaptic connection.
Assawachananont et al., 2014	Organoids cultured for 11–17 days are most likely to have synaptic connections. Transplanting the outer nuclear layer with a small amount of the inner core layer promotes the connection between the ONL of the donor and the INL of the recipient.
Goureau and Orieux, 2020	The purified PR precursor cells injected into the subretinal space and are more easily integrated with the recipient neurons.
Kegeles et al., 2020b	Adding Forskolin, an activator of adenylate cyclase, on the first day of organoid culture improves the efficiency of visual field induction.
Chen et al., 2016	70% of organoids are rod cells.
Pan et al., 2020	COCO promotes the generation of PR precursor cells.
Völkner et al., 2016	Inhibitors of the Notch pathway can promote cone cells when added in the early stage, and can promote rod cells when added in the later.
Gagliardi et al., 2018	Magnetically activated cell sorting (MACS) can separate CD73(+) CRX + photoreceptor cells.
Lakowski et al., 2015, 2018	CD73(+) marked mouse-derived PR precursors will differentiate into rod cells after implantation in the subretinal space, and will be marked by Recoverin. CD73(+) can screen mouse-derived rod cells, but it is not effective for human-derived cells. Cell marker CD29 (–)/SSEA-1 (–) can screen human-derived photoreceptor cells.

### Extraction and Transplantation of Photoreceptor Cells

For retinal degenerative diseases caused by the loss of photoreceptors, subretinal transplantation of RO sheets and PRCs or even RPCs can be performed to compensate for the function of degraded PRCs, which acts as a type of patch (Singh and Nasonkin, 2020). Pearson et al. (2012) found that an integrated graft can provide vision in mice with retinal degeneration under low-light conditions and can project signals to the V1 area of the brain, which is mainly responsible for visual processing.

Studies have found that the development of human–derived ROs after 30–70 days of culture *in vitro* is equivalent to that of embryos at 8–14 weeks. At the same time, the implanted organoids can continue to develop, differentiate, and constantly integrate information with the recipient’s retina. McLelland et al. (2018) showed that connection of the donor lamina to the receptor’s inner plexiform layer indicates the establishment of synaptic connections. Assawachananont et al. suggested the use of mouse–derived ROs cultured for 11 to 17 days for transplantation. These ROs have not matured, but they make the most efficient synaptic connections with the recipient (Assawachananont et al., 2014). Studies have found that transplantation of organoid sheets is not an effective therapy because of their unorganized structure and complex cell types. Goureau and Orieux (2020) suggested that direct injection of purified mouse–derived photoreceptor precursor cells into the subretinal space can promote the production of a single cell type in the graft and facilitate the exchange of information and substances with receptor neurons, resulting in more efficient and accurate replacement therapy. Therefore, PRCs should be extracted from organoids before transplantation into the subretinal space. Additionally, Singh et al. found that if PRCs and neurons in mouse–derived ROs were transplanted together, the neurons connected to RGCs could still survive even if the PRCs subsequently underwent apoptosis (Lin and Peng, 2013). Assawachananont et al. (2014) suggested that if the outer nuclear layer to be transplanted in the mouse–derived RO contains a small amount of the inner nuclear layer, connections between the donor outer nuclear layer and the recipient’s inner nuclear layer will be promoted. Kegeles et al. (2020b) added forskolin, an adenylate cyclase activator, on the first day of mouse ESC–derived RO culture, and the induction efficiency of the visual field was significantly improved.

Some studies have also used embryonic stem cells and iPSCs from Nrl–green fluorescent protein mice to induce ROs and evaluate the development of rod cells *in vitro*. The results showed that up to 70% of the cells in ROs were rods (Chen et al., 2016). The addition of COCO as an auxiliary supplement to medium has also been shown to promote the production of human–derived photoreceptor precursor cells and, in the long term, leads to more cones than rods (Tanaka et al., 2015). Nakano et al. (2012) found that Notch signaling pathway inhibitors is able to accelerate the generation of human–derived photoreceptors. Völkner et al. found that it can also control the differentiation trend of mouse–derived precursors. If a Notch pathway inhibitor is added in the early stage of differentiation, the generation of cones will increase. If the inhibitor is added in the late stage, the growth of rod cells will be promoted (Völkner et al., 2016).

Labeling PRC–specific reporter genes such as *CRX* also plays an important role in separating PRCs of ROs for subsequent transplantation. Gagliardi et al. showed that the *mCherry* gene is specifically expressed in all *CRX*(+) PRCs, and surface antigen marker CD73 is specifically expressed in *mCherry*(+) cells, which provides a feasible method to efficiently collect *CRX*(+) PRCs. Magnetic activated cell sorting can thus be used for targeted separation of *CRX*(+) PRCs with the specific surface antigen marker CD73 (Gagliardi et al., 2018). CD73(+) is a rod-specific marker for mouse-derived ROs that can be labeled with rhodopsin, but its specificity is low for human–derived ROs. Lakowski et al. (2015, 2018) suggested that a higher proportion of PRCs can be obtained from human–derived ROs by identifying CD29(–)/SSEA-1(–) cells. There is also research on targeted screening of mouse–derived CD73(+) CD24(+) CD133(+) CD47(+) CD15(–) photoreceptor precursors, which will then differentiate into functional rods that can be labeled by Recoverin (Lakowski et al., 2015).

The differentiation and maturation processes of PRCs of ROs from different sources obviously differ (Hallam et al., 2018). Therefore, to reduce the heterogeneity of culture, the currently used protocol, serum-free floating culture of embryoid body–like aggregates with quick reaggregation, has been improved, and the modified protocol called High Efficiency Hypoxia Induced Generation of Photoreceptors in ROs protocol was used so that the development of mouse–derived ROs and their PRCs could be highly representative of the retina *in vivo* (Chen et al., 2016). Labeling a specific gene of PRCs, *CRX*, with the reporter gene *tdTomato* can help to track the differentiation process of human–derived cones and rod cells. This alteration will not affect the differentiation of ROs. Additionally, the fluorescence intensity of the *tdTomato* gene is consistent with that of flow cytometry; therefore, the degree of differentiation of precursor cells can be quantified (Pan et al., 2020).

At present, the transplantation therapy of PRC is more mature than that of ganglion cells. It has been proven to be an alternative treatment for retinal degenerative diseases and provide scotopic vision. This is achieved through a successful synaptic connection with the inner plexiform layer of the receptor. However, the current difficulty lies in the complex and disordered structure of the organoid lamellar tissue itself, which affects the therapeutic effect. Therefore, the better solution is to separate and extract the PRCs to a certain extent. Using *CRX* reporter gene and cell markers such as CD73, PRCs can be separated more accurately. At the same time, COCO and Notch signal inhibitors can control the relative proportion of cone and rod cells in ROs, indirectly making the transplant more precise. On the other hand, the High Efficiency Hypoxia Induced Generation of Photoreceptors in ROs protocol and *tdTomato* reporter gene can reduce the heterogeneity between PRCs from different sources. The improved protocols and research findings mentioned in this section are separately summarized in Tables 1, 2.

## Discussion

Because ROs have highly similar structures and cell types to retinas, the RO model will be an ideal choice for studying the mechanisms of diseases, developing efficient drugs, and examining organ transplantation. However, because of their low yield, long culture time, and high heterogeneity of products under different culture conditions, the application and translation of ROs are restricted at this stage.

Currently, methods such as computer deep learning algorithms, multimodal imaging, and immunohistochemistry can be used to predict the direction of differentiation, monitor development and even metabolic processes in real time, and perform quantitative evaluations without destroying the structure of organoids. This has allowed the development process of ROs to be categorized into three stages, which greatly reduces the heterogeneity among products under different conditions. Regarding the problem of low yield, existing research has improved the efficiency of organoid culture by dividing the original product in advance and improving the utilization of cells. Controlling cell density, the use of pure chemical culture media, and the addition of retinal developmental substances to the culture medium can promote organoid differentiation, and the development of related biological scaffold materials will undoubtedly shorten the maturation process of ROs.

Currently, studies have shown that ganglion cells can be transplanted into the corresponding ganglion cell layer, and the transplanted cells can compensate for the loss of function. However, the implanted ganglion cells have a relatively short lifespan, and there is also the problem that the number of ganglion cells and the length of axons available in the traditional RO protocol are not sufficient to connect to the visual pathway. Some studies have suggested that the combination of three-dimensional and two-dimensional protocols can promote the expansion of ganglion cells. Additionally, as the survival time of ganglion cells increases, the ability of ganglion cells to grow axons in organoids increases, and axon functions become more mature. The addition of Muller cells can increase the short lifespan of RGCs in culture and promote the functional recovery of RGCs that have degenerated because of disease after transplantation into the receptor. This effect may be related to the transcription factor ATOH7, which promotes RGC development, but it may decrease the number of progenitors in organoids, thus reducing the number of PRCs during subsequent development.

The current technology can also provide scotopic vision for mice with retinal degeneration via transplantation of RO sheets or subretinal cells. Synaptic connections can be verified when the inner plexiform layer of the receptor is connected to the donor, and these connections will not degenerate over time. In addition, the use of early-stage ROs is recommended to establish connections more effectively. A higher proportion of cone or rod cells can be obtained using photoreceptor precursor cells extracted from ROs, adding a Notch signaling inhibitor to RO culture at different stages, or targeted isolation based on cell markers, resulting in more accurate and efficient cell replacement therapy. The existing technology can be used to develop photoreceptors that are similar to those in retinas and assess the degree of cell differentiation. Optical coherence tomography, visual dynamics tests, immunohistochemistry, and other methods can be used to determine the effect of transplantation.

As mentioned above, the latest studies have made progress in solving the problems of insufficient yield, large heterogeneity, and long process of ROs culture. However, when the ganglion cells or PRCs produced from organoids are transplanted into the body, it is still unknown whether they can create long-term clinical functional connections with visual pathways. Therefore, in order to apply the cultivated organoids to the clinical practice and benefit the patients, there is still a long way to go.

## Author Contributions

XL and XW contributed conception and design of the review. LZ and FT provided critcial revisions to the content. All authors contributed to the article and approved the submitted version.

## Conflict of Interest

The authors declare that the research was conducted in the absence of any commercial or financial relationships that could be construed as a potential conflict of interest.
