# Corrigendum: Retinal Organoids: Cultivation, Differentiation, and Transplantation

**DOI:** 10.3389/fncel.2021.810268

**Published:** 2021-12-02

**Authors:** Xuying Li, Li Zhang, Fei Tang, Xin Wei

**Affiliations:** ^1^Department of Ophthalmology, West China Hospital, Sichuan University, Chengdu, China; ^2^Department of Ophthalmology, Shangjin Nanfu Hospital, Chengdu, China

**Keywords:** retinal organoid, stem cell, retinal ganglion cell, photoreceptor cell, replacement therapy

In the original article, there was a mistake in Figure 1 as published. The **Figure B** labeled as Hyperspectral imaging is the Fluorescence Lifetime Imaging Microscopy figure, and the **Figure C** labeled as Fluorescence Lifetime Imaging Microscopy is the Hyperspectral imaging figure. The corrected Figure 1 appears below.

**Figure 1:**
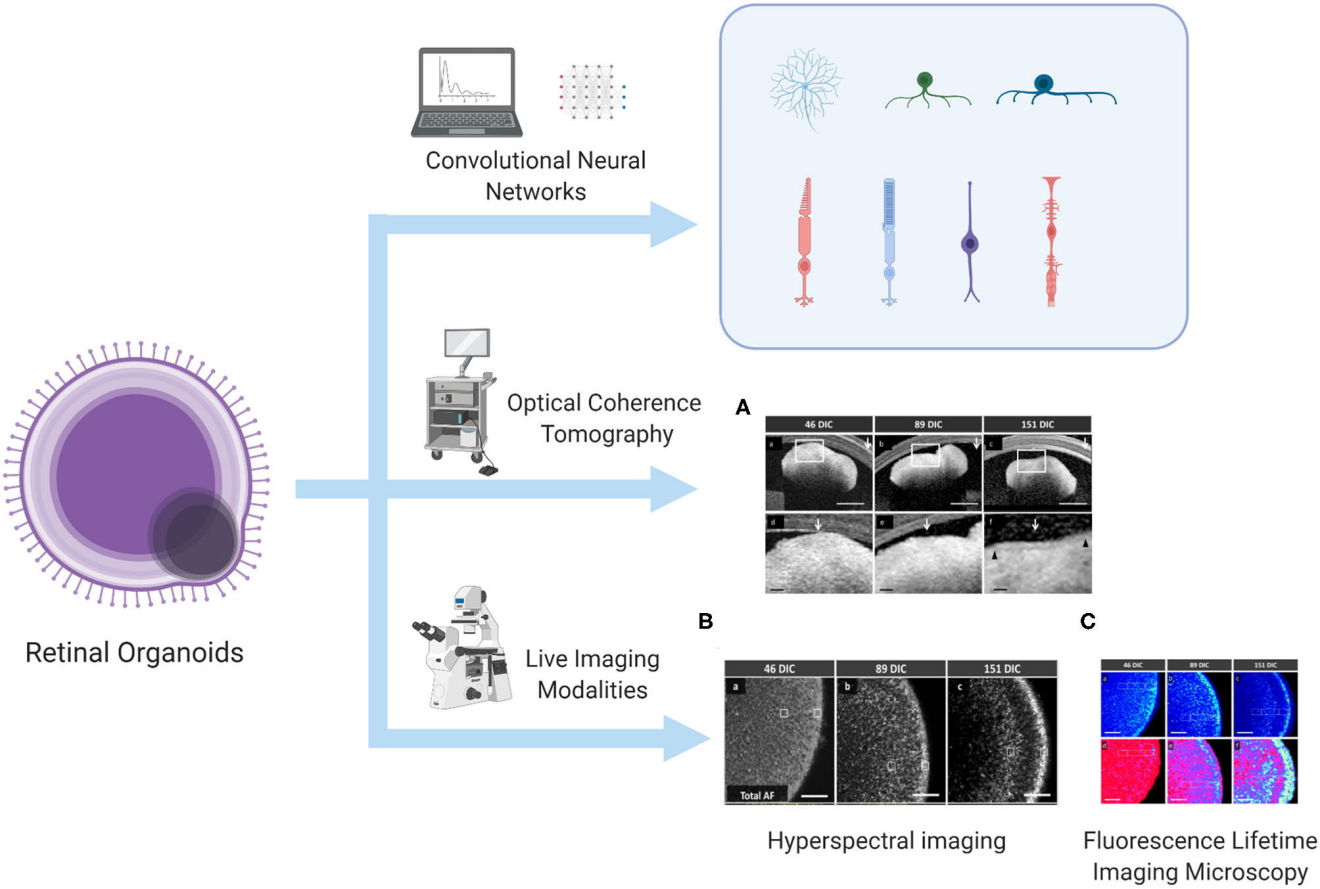
Algorithm to predict and identify organoid differentiation, and real-time imaging modalities to monitor the metabolic status of the lamellar structure of ROs. This illustration is created by Biorender.com **(A)** OCT image, **(B)** HSpec image, and **(C)** FLIM image are reprinted with permission from ref (Browne et al., 2017). Copyright © 2017 Browne AW. et al.

The authors apologize for this error and state that this does not change the scientific conclusions of the article in any way. The original article has been updated.

## Publisher's Note

All claims expressed in this article are solely those of the authors and do not necessarily represent those of their affiliated organizations, or those of the publisher, the editors and the reviewers. Any product that may be evaluated in this article, or claim that may be made by its manufacturer, is not guaranteed or endorsed by the publisher.

